# Effect of attachment flash on clear aligner force delivery: an in vitro study

**DOI:** 10.1186/s12903-024-04284-9

**Published:** 2024-05-07

**Authors:** Marisa Kiong, Asma Ashari, Nurul Syahira Mohamad Zamani, Reuben Axel Wee Ming How, Rohaya Megat Abdul Wahab, Alizae Marny Fadzlin Syed Mohamed, HeeJeong Jasmine Lee, Mohd Hadri Hafiz Mokhtar

**Affiliations:** 1 Department of Family Oral Health, Faculty of Dentistry, Universiti Kebangsaan Malaysia, Jalan Raja Muda Abdul Aziz, 50300 Kuala Lumpur, Malaysia; 2 Department of Electrical, Electronic and Systems Engineering, Faculty of Engineering and Built Environment, Universiti Kebangsaan Malaysia, 43600 Bangi, Selangor Malaysia; 3 Tristar Aligner Materials, 7-32 Berjaya Times Square, 1 Jalan Imbi, 55100 Kuala Lumpur, Malaysia; 4 College of Information and Communication Engineering, Sungkyunkwan University, Suwon, 16419 South Korea

**Keywords:** Attachment, Clear aligner, Flash, Force, Image processing

## Abstract

**Background:**

The introduction of auxiliaries such as composite attachment has improved the force delivery of clear aligner (CA) therapy. However, the placement of the attachment may give rise to a flash, defined as excess resin around the attachment which may affect CA force delivery. This in vitro study aims to determine the differences in the force generated by the attachment in the presence or absence of flash in CA.

**Materials and methods:**

Tristar Trubalance aligner sheets were used to fabricate the CAs. Thirty-four resin models were 3D printed and 17 each, were bonded with ellipsoidal or rectangular attachments on maxillary right central incisors. Fuji Prescale pressure film was used to measure the force generated by the attachment of CA. The images of colour density produced on the films were processed using a calibrated pressure mapping system utilising image processing techniques and topographical force mapping to quantify the force. The force measurement process was repeated after the flash was removed from the attachment using tungsten-carbide bur on a slow-speed handpiece.

**Results:**

The intraclass correlation coefficient showed excellent reliability (ICC = 0.96, 95% CI = 0.92–0.98). The average mean force exerted by ellipsoidal attachments with flash was 8.05 ± 0.16 N, while 8.11 ± 0.18 N was without flash. As for rectangular attachments, the average mean force with flash was 8.48 ± 0.27 N, while 8.53 ± 0.13 N was without flash. Paired *t*-test revealed no statistically significant difference in the mean force exerted by CA in the presence or absence of flash for both ellipsoidal (*p* = 0.07) and rectangular attachments (*p* = 0.41). Rectangular attachments generated statistically significantly (*p* < 0.001) higher mean force than ellipsoidal attachments for flash and without flash.

**Conclusion:**

Although rectangular attachment generated a significantly higher force than ellipsoidal attachment, the force generated by both attachments in the presence or absence of flash is similar (*p* > 0.05).

## Background

Improvement in dental and facial appearance is usually the leading reason for seeking orthodontic treatment [1, 2]. There has been an upsurge in demand for more comfortable and aesthetic orthodontic appliances, especially among the adult population, and this causes clear aligner (CA) therapy to gain popularity [3]. CA therapy involves using thermoformed plastic aligners that are replaced sequentially to correct malocclusions.

Literature evidence demonstrated that this “invisible” aligner is not only more visibly pleasing [4, 5], but it also provides a less painful alternative to conventional orthodontic appliances [6–8] as well as better oral health-related quality of life (OHRQoL) [7]. CA therapy also facilitates oral hygiene practices [9] and contributes to better periodontal health [10–12]. Apart from that, the incidence and severity of white spot lesions (WSLs) [13, 14] and root resorption [15, 16] is reduced compared to fixed orthodontic appliances.

Despite various reported benefits of CA therapy, evidence of its efficiency and effectiveness is still lacking [17–21]. Various studies were conducted to compare the force delivery properties of the CA, and the bodily movement and torque were found to be the most demanding movement to be achieved purely by CA without any further modifications [22–24].

The introduction of auxiliaries such as composite attachment has revolutionised CA therapy by improving force delivery onto teeth and generating the required force couple for bodily movement and torque [25–29]. Studies have shown that complex tooth movements such as incisor torque, rotation, and molar distalisation can now be achieved in CA treatment by using auxiliaries like composite attachment [18, 30].

Despite the benefits provided by the application of attachment, it inevitably may give rise to flash during the bonding of the attachment due to the flowable nature of the composite resin. Flash is an excess resin that may be formed around the attachment during the bonding of resin onto teeth.

The initial bonding of the attachment is imperative and needs to be accurately placed on the tooth surface as any deviation in the attachment’s position can lead to improper tooth movements and inadequate force intensity which will jeopardise the fitting of the subsequent CA [31]. Weckmann et al. [31] investigated the influence of attachment bonding protocol on the precision of attachment in aligner treatment. The authors found that bonding protocol does influence the precision and amount of flash produced. It was reported that the use of high viscosity composite without perforation in the attachment reservoir was the most inaccurate while the use of low viscosity composite without perforation produced the largest amount of flash. The protocol with the attachments made by the two-phase procedure with high-viscosity composite seemed to be the most precise and produced the least amount of excessive composite area bonded around the attachment. In this protocol, the template with composite resin was placed on the model but was not cured immediately. The template was removed to allow the excess composite to be removed first before reinserting the template on the model and light cured.

Another available literature found is on the flash formed during the bonding of brackets in the fixed appliances therapy. This flash can be present around the borders between conventional brackets and enamel upon bonding of brackets. The clinician will need to remove the excess resin after placement of the bracket on the tooth during the bond-up process before curing the adhesive. The excess adhesive in fixed appliances if not removed completely, can act as a mechanical irritation to the gingiva, especially on teeth where the distance to the gingiva is small and bacteria will readily colonise the surface of rough composites [32], leading to plaque retention and increase in the incidence of WSLs [33, 34]. Thus, it is essential to remove the flash thoroughly to reduce the incidence of plaque accumulation and WSLs.

Although it is preferred that thorough removal of the adhesive flash around the brackets is carried out, it is time-consuming and technique-sensitive [35]. The process of flash clean-up during the bonding of fixed appliances tends to prolong the appointment. This is because the composite used has the same colour as the enamel, making detecting the remaining composite challenging especially gingival to the bracket area [36]. Although the removal of adhesive flash may require an additional few seconds per tooth, cumulatively, the time saved from carrying out this step may add up to be clinically significant when it involves both arches [35].

Thus far, there is no study on the effect of attachment flash removal in CA therapy. Typically, rotary instruments equipped with tungsten carbide finishing bur are used to remove this flash. Since similar tungsten carbide bur is used to remove residual resin left on the enamel after bracket debonding in fixed appliances [37–39], a similar effect to the enamel may be anticipated for the removal of attachment flash. Studies have reported that there is always a possibility of iatrogenic damage to the enamel from the removal of adhesive after debonding of orthodontic brackets which causes irreversible damage to the tooth [37, 40]. According to the literature, regardless of the techniques or instruments used, scratches and damage to the enamel are unavoidable when removing remnant adhesive after debonding [39, 41, 42]. The time consumed for the removal of residual resin will be lengthy when performed with low-speed rotary instruments [38, 43]. This translates into longer chairside time [43]. Though the use of a high-speed drill may be faster, it is associated with detrimental enamel loss [44]. The high-speed bur used for the removal of adhesive remnants caused deeper scratches compared to the bur at a slow speed [38]. The enamel roughness caused by this step may also lead to plaque accumulation [45]. Moreover, patients can experience tooth sensitivity due to enamel loss during debonding [46, 47]. Hence, similar iatrogenic enamel loss and roughness are possible when removing a flash from attachment bonding in CA therapy.

Since the CA is closely adapted to the attachment to direct the force onto the tooth to achieve movement, the presence of flash around the attachment may affect the force delivery by the CA. There is substantial data in the literature regarding the flash in orthodontic fixed appliances. However, evidence of the impact of flash on the attachment towards force expressed by CA and its influence on tooth movement is still lacking.

The study aims to determine the difference in the force generated by the CA in the presence or absence of flash on ellipsoidal and rectangular attachments. The null hypothesis was that there was no significant difference in the force generated by the CA in the presence or absence of flash on ellipsoidal and rectangular attachments.

## Materials and methods

This study was an in vitro study carried out in the Orthodontic Unit, Department of Family Oral Health, Universiti Kebangsaan Malaysia (UKM). Ethical approval was obtained from the Universiti Kebangsaan Malaysia Research Ethics Committee (JEP-2021–410).

### Sample size

The sample size was calculated based on a significance level of 0.05 and 80% power to detect a clinically meaningful difference for the force exerted by CA with a standard deviation of 0.43 N [48]. The sample size calculation by G*Power 3.1.9.7 software (Institute for Experimental Psychology, Dusseldorf, Germany) gave a total of 17 subjects per group.

### Resin models fabrication

Resin models and CAs were constructed in Whitesmile Clear Laboratory (Visivest Corporation (M) Sdn. Bhd., Kuala Lumpur, Malaysia). An anonymous patient’s maxillary arch with fairly aligned teeth was scanned using an intraoral scanner (CAMEO Intraoral Scanner, Aidite Technology Co., Ltd, Hebei, China). The Standard Triangle Language (STL) file obtained from the intraoral scanning was uploaded to the 3Shape OrthoAnalyzer software (TRIOS 3Shape A/S, Copenhagen, Denmark). The 3-dimensional (3D) digital model was sectioned to remove most of the teeth, except for the maxillary right central incisor and two maxillary premolars. This was done to make it easier to fit the aligner and reduce any potential interferences caused by other teeth. Removal of the interferences will allow better quantification of the force exerted on a single tooth.

This STL file of the modified model was further edited in the Autodesk Meshmixer version 3.5 software (Autodesk, Inc., California, USA) before being exported to Chitubox version 1.9.4 software (Chitubox, Guangdong, China) to prepare for 3D printing. The Uniz Slash 2 3D printer (Uniz Technology LLC, California, USA) along with Uniz zDental Model photopolymer resin (Uniz Technology LLC, California, USA) was used to print 34 resin models from the digital models. The printing and post-curing process strictly adhered to the manufacturing instructions. The printed models were carefully inspected for any errors during the printing process. If any issues were detected, the models were reprinted to ensure high quality.

### Attachment design

**Fig. 1:**
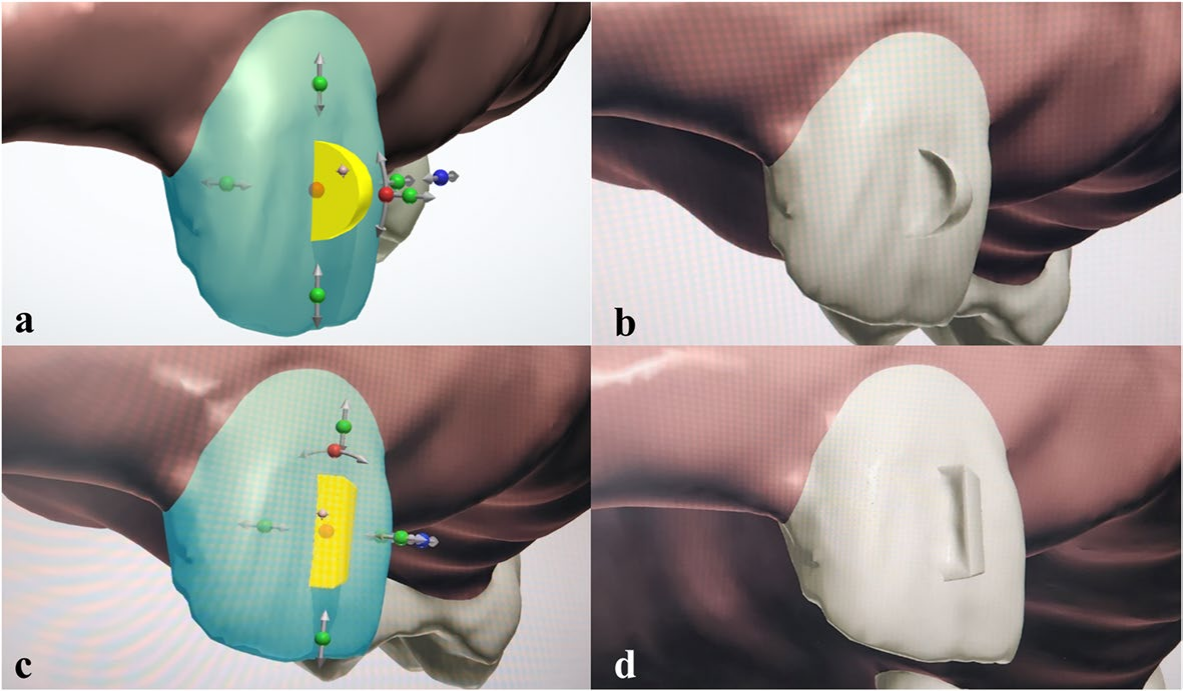
Distal surface of the ellipsoidal (**a** and **b**) and rectangular (**c** and **d**) attachments were sculpted

This study utilised two types of attachments, ellipsoidal and rectangular. These attachments with bevels on the distal surfaces were designed for 2.5 degrees of rotation activation of the maxillary right central incisor. The attachments were virtually placed on the midfacial surface of the crown of the maxillary right central incisor using the 3Shape OrthoAnalyzer software (3Shape A/S, Copenhagen, Denmark). The resin models with ellipsoidal and rectangular attachments were 3D printed out with Uniz Slash 2 printer (Uniz Technology LLC, California, USA) for the attachment template fabrication. For the resin models to be used for CA fabrication, the attachments were virtually sculpted further on the distal surface of the attachment making an indentation before another 34 resin models with ellipsoidal and 34 resin models with rectangular attachments were printed out (Fig. 1). As the CAs are made using a suck-down method on top of the resin models, the sculpted area, which appears as an indentation creates a thicker part of the CA in that area. Therefore, the thicker part of CA creates forces when it is seated clinically in the patient’s mouth where the attachments are not sculpted. This is the basis of how CA works and creates movements in teeth.

### Clear aligner and attachment template fabrication

Tristar Trubalance (Visivest Corporation (M) Sdn. Bhd., Kuala Lumpur, Malaysia) aligner sheets with a thickness of 0.76 mm were used to manufacture the CAs. Tristar is a multi-layered composite material that combines polyurethanes and polyethylene terephthalate glycol (PET-G). This material possesses high impact strength and flexibility allowing it to deliver consistent force throughout the application period.

Thirty-four active aligners of ellipsoidal and rectangular attachments, each were fabricated using a pressure forming machine (Ministar S®, Scheu Dental, Iserlohn, Germany). The sculpted region of the attachment on the resin model which was indented caused a thicker material of CA over the attachment region when CA was pressure formed. This exerted extra force over the attachment to derotate the specific tooth. In this study, the area of interest was the sculpted area of the attachment.

Attachment templates were used for bonding the attachment onto the resin models. Tristar Attachment Template sheet (Visivest Corporation (M) Sdn. Bhd., Kuala Lumpur, Malaysia) which is a 0.3 mm thickness mono-layered template material was used to fabricate the templates using a similar technique as the CA utilising pressure forming machine (Ministar S®, Scheu Dental, Iserlohn, Germany).

### Attachment placement

Thirty-four resin models were divided into two groups, 17 each for ellipsoidal and rectangular attachments. These resin models underwent attachment bonding following the manufacturer’s instructions using 37% phosphoric acid (Meta Etchant, Meta Biomed Co., Ltd, Chungcheongbuk-do, Republic of Korea), bonding agent (Meta P & Bond, Meta Biomed Co., Ltd, Chungcheongbuk-do, Republic of Korea), and composite resin (Filtek Z350 XT, 3 M ESPE, Minnesota, USA). The exact amount of composite was filled into the well of the attachment template for ellipsoidal (0.02 g) and rectangular attachments (0.01 g), and the template was pressed onto the resin model surface and light cured (SDI Radii-cal LED curing light, 1200 mW/cm^2^, SDI Limited, Victoria, Australia). The attachments were made with composite resin in the A1 shade, which provided a higher contrast against the yellow resin models. This made it easier to remove any excess material later on.

### Orthodontic force measurement with pressure-sensitive film

Stress can be measured using destructive and non-destructive techniques. In our work, pressure mapping was achieved via a non-destructive technique [49]. The method for force measurement was adopted from Zamani et al. [50]. The orthodontic force generated by the CA was measured using the pressure-sensitive film (Fuji Prescale Film, Fujifilm Corporation, Tokyo, Japan). The film consists of two sheets: A- and C- film. When used, the coated surfaces of the two films were placed facing each other. The combined width of the two films is 90 ± 5 μm. These polyester films are designed to be pressure-sensitive with a reduced thickness that minimises the interface contact while effectively distributing and quantifying direct force. When the pressure was applied to the sheets, the micro-encapsulated colour-forming material on the A-film was broken down and reacted with the colour-developing material on the C-film, thereby generating colour [51, 52]. As a result, the colour density ranging from pale pink to magenta was developed, corresponding to the pressure level applied.

**Fig. 2:**
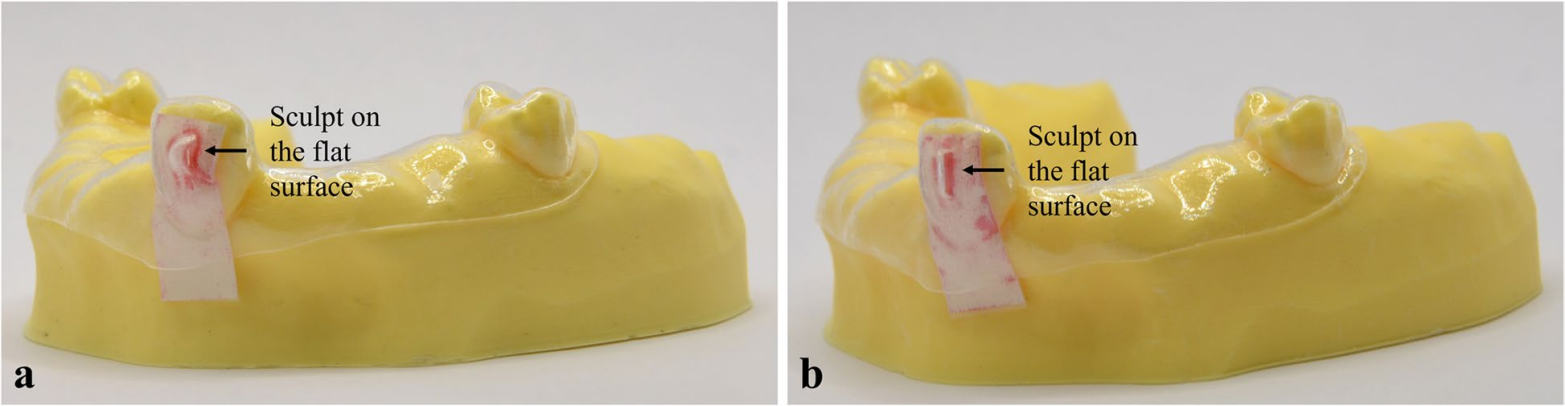
Sculpted area of ellipsoidal (**a**) and rectangular (**b**) attachments

The Super Low Pressure (LLW) film for the measurement of force ranging from 0.5–2.5 MPa was used for this study. The two A- and C-films were cut to the approximate size of the labial surface of the right central incisor and put together with their active, matte surfaces in contact. The pressure films were then inserted between the labial surface of the resin models with attachment and CA, resulting in pressure stain samples (Fig. 2). The film responded to the pressure by turning to different shades of magenta. The colour intensity was proportionate to the amount of pressure applied.

**Fig. 3:**
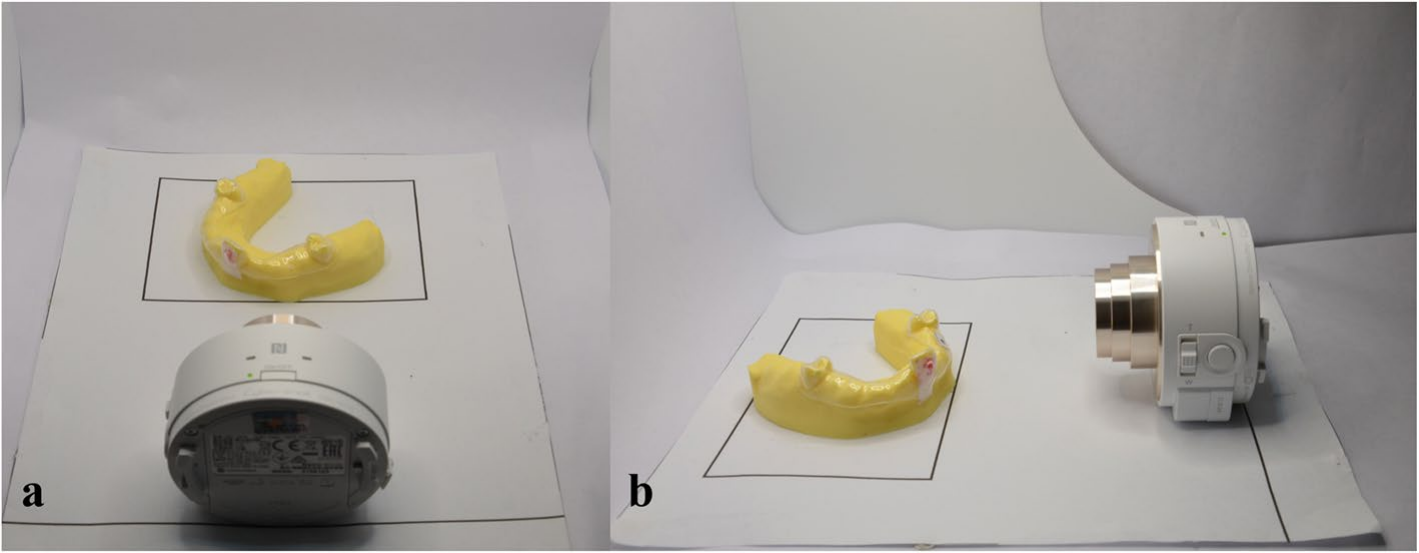
Image acquisition in photo studio box from frontal (**a**) and side (**b**) views

This study’s primary focus was on the sculpted area of the attachment. Thus, the images of the colour density produced on the films at the sculpted area of the attachments were captured immediately in a standardised manner. A photo studio box and a digital microlens camera (Cybershot DSC-QX10, Sony, Tokyo, Japan) were used to capture the images, which were wirelessly connected to a smartphone through the Imaging Edge mobile application to facilitate digital image acquisition of all the films (Fig. 3). The ambience light in the mini studio and the placement of the camera and target object was kept constant throughout the experimental procedure to ensure that the image acquisition was consistent.

**Fig. 4:**
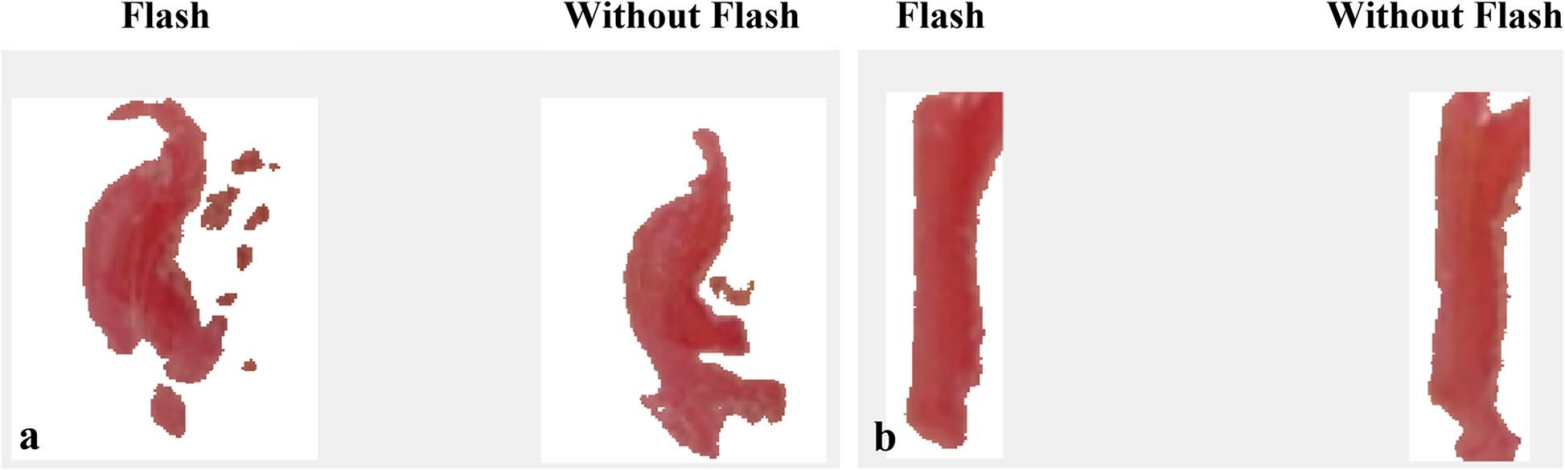
Cropped and segmented images of ellipsoidal (**a**) and rectangular (**b**) attachments

The colour density of stained films was processed using tailored coding via the image processing toolbox in MATLAB (MathWorks, Massachusetts, USA). The images obtained were processed using a topographical pressure mapping system and the intensity level of the colour was converted into force, where all values had been calibrated (Fig. 4). Prior to that, the images were cropped to the appropriate region to be converted into different colour space called L*a*b (Lightness, channel a and channel b). This colour space was chosen due to the most prominent pixel of the stained film.

The cropped region was processed to segment the foreground region, the stained film, using image clustering. Clustering was an unsupervised machine learning technique where the unlabelled grouped (foreground and background) regions were implemented with the three conditions (masks). This condition required the user to select the desired mask. The selected mask was mapped with the original image and converted into HSV (hue, saturation, value) colour space for intensity visualisation before topographical mapping. A detailed prescription of the system was explained in the paper by Zamani et al. [50].

The flash around the attachments on the resin models was removed using tungsten carbide finishing bur attached to a slow-speed handpiece. Complete flash removal was confirmed once there was no excess A1 shade composite resin material at the attachment margin while ensuring no yellow resin was removed from the models. Force measurement steps using Fuji Prescale pressure-sensitive film and topographical pressure mapping system were repeated on resin models without flash.

### Method error

To ensure uniformity, the attachment bonding and the removal of flash were carried out by the same experienced researcher calibrated by an orthodontist with more than five years of experience with CA.

To prevent inaccurate force measurement due to force decay from multiple times CA insertion onto the models, new identical CAs were used each time for force measurement in models with and without flash.

Intra-examiner reliability was assessed by reprocessing ten samples from each group using the topographical pressure mapping system at least two weeks later, and the measurements were compared to the mean of the initial measurement.

A random error was minimised by taking measurements of the force twice and calculating a mean force for both ellipsoidal and rectangular attachments.

### Statistical analysis

The data collected were subjected to computerised statistical analysis using SPSS Version 26 (IBM Inc., New York, USA). The normality assumption of the data was measured using the Shapiro–Wilk test. Since the variables were normally distributed and there were no significant outliers, paired *t*-test analysis was used to determine any statistical difference between the force generated by attachments in the presence and absence of flash. In addition, an independent *t*-test was used to detect the statistical difference between the force of ellipsoidal and rectangular attachments. The level of significance was set at *p* < 0.05.

Intra-examiner reliability for force measurement was assessed using intraclass correlation coefficient (ICC). ICC revealed excellent intra-examiner reliability of 0.96 (95% CI = 0.92–0.98).

## Results

Table 1 shows the mean force measured for ellipsoidal and rectangular attachments in the presence and absence of flash. The average mean force exerted by ellipsoidal attachments with flash was 8.05 ± 0.16 N, while 8.11 ± 0.18 N was without flash. As for rectangular attachments, the average mean force with flash was 8.48 ± 0.27 N, while 8.53 ± 0.13 N without flash. Paired *t*-test revealed no statistical difference (*p* > 0.05) in the mean force exerted by attachments in the presence or absence of flash for both ellipsoidal (*p* = 0.07) and rectangular attachments (*p* = 0.41). Rectangular attachments generated a significantly higher mean force than ellipsoidal attachments for both flash and without flash (*p* < 0.001).

**Table 1:** Force for ellipsoidal and rectangular attachments

Attachment Shape	Flash			Without Flash			*p*-value
	Range (N)	Average mean force (N)	SD	Range (N)	Average mean force (N)	SD	
Ellipsoidal attachment (*n* = 17)	7.75–8.37	8.05	0.16	7.74–8.36	8.11	0.18	0.07
Rectangular attachment (*n* = 17)	8.07–8.82	8.48	0.27	8.37–8.79	8.53	0.13	0.41
*p*-value		< 0.001^a^			< 0.001^a^		

n, number; N, Newton; SD, standard deviation

^a^Significant *p*-values

**Fig. 5:**
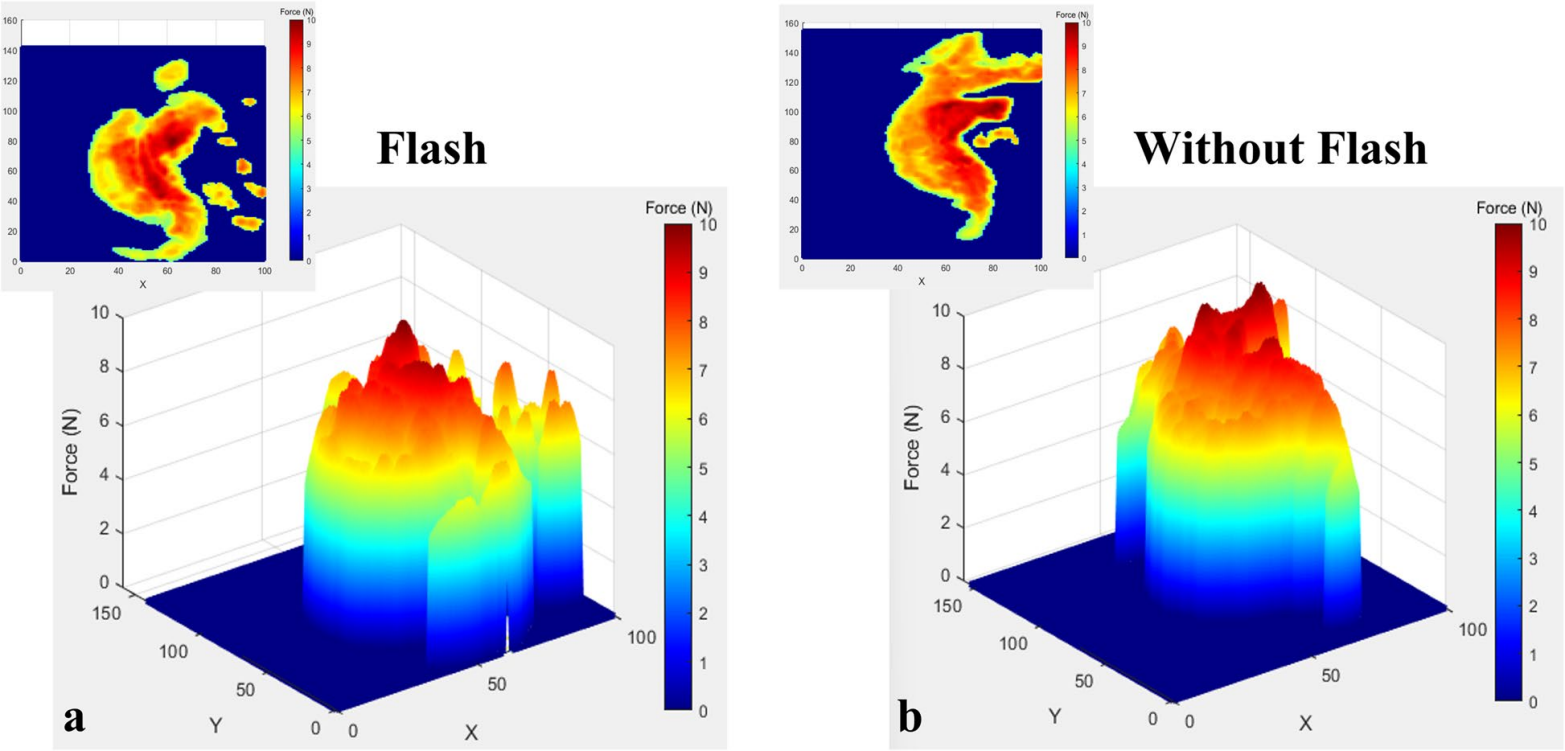
3D topographical mapping of ellipsoidal attachment with flash (**a**) and without flash (**b**)

**Fig. 6:**
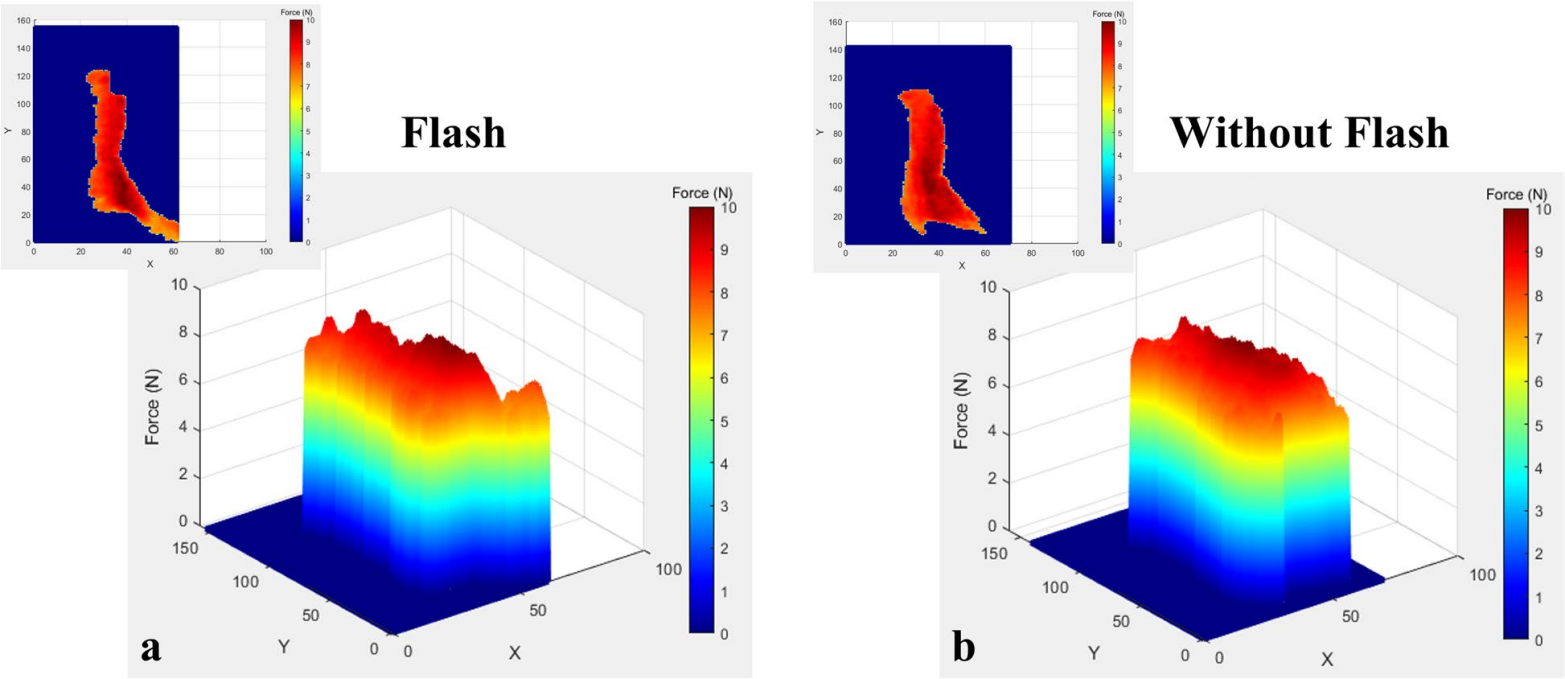
3D topographical mapping of rectangular attachment with flash (**a**) and without flash (**b**)

Figures 5 and 6 illustrate the 3D topographical mappings of the force distribution of ellipsoidal and rectangular attachments with and without flash, respectively. The colour signified the level of force exerted by the attachment. The colour ranged from blue colour, indicating low force to red colour, indicating high force. It has been observed that the force was concentrated at the centre of the sculpted area as the centre shows a more intense shade of red while the periphery of the attachment shows a yellow to orange shade, indicating a lower force.

The 3D topographical maps also clearly demonstrated similar results to statistical analysis where the rectangular attachment exerted a significantly higher force than the ellipsoidal attachment for both with flash and without flash. This was evident in the larger area of red and more intense shade of red displayed in the 3D maps of the rectangular attachment.

## Discussion

To our knowledge, this current study is the first study to investigate the effect of attachment flash on the force exerted by CA. It was found that there was no significant difference in the forces exerted by CA in the presence or absence of attachment flash. This is an interesting result as attachment flash is commonly considered a potential interference that could affect the CA force delivery. The mean force was slightly higher in the absence of flash for both ellipsoidal and rectangular attachments. It was hypothesised that the CA could sit better in the absence of flash and exert force on the tooth, though not significantly different from the force in the presence of flash.

The in vitro study by Zamani et al. [50] reported a similar magnitude of the force in active aligners with ellipsoidal attachment generating an average mean force of 6.18 N and 6.34 N for rectangular attachment. Notwithstanding, the authors used both active and passive CAs. Passive aligners were used to identify the initial force upon seating the aligners. The net force exerted on a single CA attachment was 1.3–1.4 N, after deducting the initial seating force. In this present study, the initial seating force was assumed to be constant, thus the force obtained from this study between with and without flash was compared to determine any force difference.

Barbagallo et al. [53] utilised a similar pressure-sensitive film to clinically measure the force exerted by 0.8 mm thickness CA programmed for 0.5 mm buccal tipping on the lingual of maxillary first premolar. The stain intensity formed on the film was integrated digitally, and the force was quantified with spectrophotometry. However, a lower mean initial force of 5.12 N was documented [53]. This lower mean force was obtained after deducting irrelevant force due to film thickness, seating force and shear pressure. The force presented in this in vivo study may be subjected to other confounding factors due to the presence of a complex oral environment. Hence, it is expected that a lower force was reported as the periodontal ligament may dissipate some of the force. Moreover, the pressure film is extremely sensitive, making it rather challenging to be used intraorally. As a result, direct application in the oral cavity might have significantly lowered the level of precision of the measurements. This study also reported the values of the force measured without any topographical mapping of the force.

Cervinara et al. [54] conducted an in vitro study utilising Fuji Prescale (Fujifilm Corporation, Tokyo, Japan) pressure-sensitive film to quantify the pressure exerted by the CA on 3D-printed resin casts. The authors removed a thin layer of the cast equivalent to the thickness of the film to eliminate unwanted additional force due to the thickness of the film. In this current study, the assumption was made that the force resulting from the film's thickness remained constant. Instead of considering the net force, the recorded force transmitted by the CA in the presence and absence of flash was compared. Similar to Zamani et al. [50], both passive and active aligners were used by Cervinara et al. [54] where the mean pressure was obtained by subtracting the mean passive pressure from the mean active pressure. Nonetheless, the authors described the pressure rather than the force exerted by CA, unlike most literature. Apart from that, a different system was used to analyse the film and the analysis resulted in a flat 2D image of pressure mapping that visualised the stain colour levels. The method employed was restricted to detecting the pressure levels in the absence of 3D visualised mapping.

Elkholy et al. [48] conducted an in vitro study using a force-measuring device comprising a six-component force/moment sensor. They found that when using 0.5 mm aligners, the median force for moving the upper right central incisor 0.25 mm in the labial and palatal direction was -7.89 N and 8.37 N respectively, which was of similar magnitude to the present study.

Although the literature presents multiple in vivo and in vitro studies of the force exerted by CA, it is difficult to compare the findings of the current study with most previous studies due to the novel methodology used. The 3D topographical mapping illustrated by this current study reveals interesting aspects regarding the distribution of the force around the attachments. The 3D maps reveal that the highest force was concentrated in the centre of the attachment and the force slowly dissipated towards the periphery. A similar distribution of force was depicted by the topographic mapping by Zamani et al. [50]. The force mapping is similar to what is clinically anticipated as the force concentration area is highest at the sculpted part of the attachment. As mentioned previously in the methodology section, the sculpted area is the area where the CA is thicker during suck-down in CA fabrication to generate sufficient force for tooth movement.

This study has also shown that the rectangular attachment exerted a significantly higher force than the ellipsoidal attachment. However, Zamani et al. [50] reported similar forces between the two attachments. It was worth noting that the authors did not carry out statistical analysis to support this statement. Similarly, Ho et al. [55] suggested that attachment shape has little effect on bodily tooth movement. Nevertheless, this area should be subjected to further study.

The presence of flash does not significantly alter the force of the CA. Therefore, it is possible to prevent iatrogenic damage of the enamel [37, 40] and tooth sensitivity [46, 47] from the removal of the flash as well as to save extra clinical time [35]. Even though it may take a few seconds to remove the flash with rotary instruments per tooth, cumulative time savings may add up and become clinically significant when considering a full-mouth application. It is desirable to shorten the chairside time as longer appointment takes up more of the patient’s time and are more expensive for the clinician [35]. Furthermore, when precise bonding of attachment is done without excessively overfilling the template's well, the flash effect is negligible. This may allow precise tooth movement, and the outcome can be predicted with greater accuracy, all without the risk of iatrogenic damage or cross-infection from aerosol produced by rotary instruments [56]. This reduces the need for instruments and the overall chairside time is shortened.

An excess amount of flash found around the conventional brackets can increase the risk of enamel demineralisation [33, 34]. However, these studies are only for conventional brackets. Ever since CA was introduced in 1998, there has been no published study on attachment flash causing demineralisation in CA. This is probably due to the very small size of the flash associated with the attachment as compared to the flash in conventional brackets. It was reported that the area of excess adhesive flash in conventional brackets is approximately 15.65 mm^2^ [57]. In contrast, the area of flash found around the CA attachment is approximately only 6.20 mm^2^ [31]. From the evidence of these two studies, it can be concluded that the size and the amount of flash around the attachment of CA is significantly smaller compared to the flash in conventional brackets. Therefore, it can be speculated that the amount of flash around the attachment in CA is insignificant to cause demineralisation. Further research is needed to prove this hypothesis.

The image processing techniques and topographical force mapping used in this current study hold the potential to serve as a valuable reference method for determining the optimal force required for tooth movement during CA treatment. The 3D topographical mapping exhibited in this study enables the distribution of the force around the attachment to be visualised. This allows a better understanding of the mechanism of attachment and helps to improve the design and placement of the attachment to achieve specific types of movement. These maps can also be used as a visual aid to communicate with patients to explain the use of attachment in their treatment [58]. Furthermore, in the realm of research, numerous studies have explored the association between orthodontic appliances and pain, with CA generally being reported as less painful based on patient-reported outcomes. However, by directly measuring the actual forces exerted, this method enables the quantification of data concerning their relationship with pain. From a commercial standpoint, this unique approach could be adopted by various aligner companies for evaluation and quality assurance purposes. Moreover, the preliminary findings of this study can serve as a basis for future clinical research, both in CA and attachment investigations, providing a valuable reference point for further advancements in the field.

### Limitations

It is important to note that the force measured in this study pertains only to a single attachment rather than the entire arch. Hence, though there was no significant difference in the force between the presence or absence of flash on a single attachment, the cumulative force difference of multiple attachments in an arch may result in a slight difference. However, based on the values, it is demonstrated to be clinically insignificant. Hence, careful interpretation of this result is warranted.

Since this is an in vitro study, the presence of alveolar bone and periodontal ligament cannot be simulated in this study. According to Proffit et al. [59], the optimal force for derotation ranges from 35–60 g which is equivalent to 0.35–0.6 N. However, in this laboratory study utilising a resin model, no tooth movement occurs with the attachment without the periodontal ligament. Consequently, the range of forces was anticipated to be significantly higher. Similar observations have been reported in previous studies utilising resin models, which also recorded higher forces [54, 60]. In addition, this study could not reproduce effects with the presence of other biological factors such as saliva, intraoral temperature, and masticatory force. Thus, the net force delivered onto teeth may be altered in a clinical setting as the mechanical properties of CA materials change in environmental factors [61].

It is worth noting that once the aligners are seated, they may generate force transmitted onto the pressure-sensitive film. Therefore, it is best to use the force obtained to compare flash and without flash, rather than relying on the net force generated.

The forthcoming study may quantify the irrelevant force such as seating and shear pressure using passive CA to obtain the net force imparted by the attachment. As this is a preliminary study, the model used in this study was obtained from a single patient. Hence it may be difficult to translate the results of this study to the general population. Future studies may obtain scans from patients with teeth in different positions to obtain a more accurate result that can be applied to the general population.

Various conditions such as shapes of flash, types of teeth, position of attachment on teeth, and the presence of adjacent teeth and multiple attachments may affect the force delivery. Thus, the effect of these variables can be investigated in future works.

In addition, the results attained from this in vitro study using the image processing technique and topographical mapping can be translated into clinical or in vivo studies as the complexity of the tooth biology and oral environment may affect the outcome. Information from clinical studies is needed to optimise treatment protocols and predict treatment outcomes accurately.

## Conclusions

This study is the first to investigate the effect of attachment flash on CA force delivery. As the use of CA becomes widespread, it is necessary to elucidate the impact of flash on the force exerted by CA. The following conclusions can be drawn from this study:

1. Both ellipsoidal and rectangular attachments with or without flash portray similar force generation in the CA. Hence, the presence of flash does not affect the CA force delivery.
2. Rectangular attachment exerted a significantly higher force than ellipsoidal attachment in the presence and absence of flash.
3. Force exerted by the CA is concentrated in the centre of the attachment, corresponding to the sculpted area.

## Data Availability

The datasets used and/or analysed during the current study are available from the corresponding author upon reasonable request.

## Abbreviations

3D: 3-Dimensional
CA: Clear aligner
HSV: Hue, saturation, value
ICC: Intraclass correlation coefficient
L*a*b*: Lightness, channel a and channel b
N: Newton
OHRQoL: Oral health-related quality of life
PET-G: Polyethylene terephthalate glycol
SD: Standard deviation
STL: Standard Triangle Language
UKM: Universiti Kebangsaan Malaysia
WSLs: White spot lesions
